# Combining the Sterile Insect Technique with the Incompatible Insect Technique: III-Robust Mating Competitiveness of Irradiated Triple *Wolbachia*-Infected *Aedes albopictus* Males under Semi-Field Conditions

**DOI:** 10.1371/journal.pone.0151864

**Published:** 2016-03-18

**Authors:** Dongjing Zhang, Rosemary Susan Lees, Zhiyong Xi, Kostas Bourtzis, Jeremie R. L. Gilles

**Affiliations:** 1 Sun Yat-sen University - Michigan State University Joint Center of Vector Control for Tropical Diseases, Zhongshan School of Medicine, Guangzhou, Guangdong Province, China; 2 Insect Pest Control Laboratory, Joint FAO/IAEA Division of Nuclear Techniques in Food and Agriculture, Vienna, Austria; 3 Department of Microbiology and Molecular Genetics, Michigan State University, East Lansing, Michigan, United States of America; University of Camerino, ITALY

## Abstract

Combination of the sterile insect technique with the incompatible insect technique is considered to be a safe approach to control *Aedes albopictus* populations in the absence of an accurate and scalable sex separation system or genetic sexing strain. Our previous study has shown that the triple *Wolbachia*-infected *Ae*. *albopictus* strain (*w*AlbA, *w*AlbB and *w*Pip) was suitable for mass rearing and females could be completely sterilized as pupae with a radiation dose of at least 28 Gy. However, whether this radiation dose can influence the mating competitiveness of the triple infected males was still unknown. In this study we aimed to evaluate the effects of irradiation on the male mating competitiveness of the triple infected strain under laboratory and semi-field conditions. The results herein indicate that irradiation with a lower, female-sterilizing dose has no negative impact on the longevity of triple infected males while a reduced lifespan was observed in the wild type males (*w*AlbA and *w*AlbB) irradiated with a higher male-sterilizing dose, in small cages. At different sterile: fertile release ratios in small cages, triple-infected males induced 39.8, 81.6 and 87.8% sterility in a wild type female population at 1:1, 5:1 and 10:1 release ratios, respectively, relative to a fertile control population. Similarly, irradiated triple infected males induced 31.3, 70.5 and 89.3% sterility at 1:1, 5:1 and 10:1 release ratios, respectively, again relative to the fertile control. Under semi-field conditions at a 5:1 release ratio, relative to wild type males, the mean male mating competitiveness index of 28 Gy irradiated triple-infected males was significantly higher than 35 Gy irradiated wild type males, while triple infected males showed no difference in mean mating competitiveness to either irradiated triple-infected or irradiated wild type males. An unexpected difference was also observed in the relative male mating competitiveness of the triple infected strain after irradiation at 28 Gy dose in small *vs* large cages, with a higher male mating competitiveness index calculated from results of experiments in the large cages. Based on these results, we consider that the male mating performance of the triple infected strain after irradiation at 28 Gy, a dose required for complete female sterility and the avoidance of population replacement, is approximately equal to that of the wild type males under semi-field conditions. Though field evaluation is required, this suggests that the triple infected strain is suitable for irradiation and release as part of a combined SIT-IIT approach to *Ae*. *albopictus* control.

## Introduction

*Aedes albopictus*, a vector of dengue and chikungunya, is one of the most invasive mosquito species and can now be found all around the world [1–3]. It was reported that in the southern province of China, Guangdong province for instance, there were more than 42,000 dengue fever cases caused by *Ae*. *albopictus* in 2014 [4]. The epidemic of vector-borne diseases indicate that traditional vector control methods are not sufficient to suppress wild type mosquito populations [4–6] and new biological control approaches, such as the sterile insect technique (SIT) and the incompatible insect technique (IIT), are being tested or applied to control the wild mosquito populations under both the laboratory and field conditions [7, 8].

Mosquito SIT and IIT both act by releasing large numbers of sterile males to compete with wild type males to mate with females in field, thereby inducing sterility in the natural female populations, leading to a decline in the target population [7–10]. Both SIT and IIT rely on several important procedures such as mass rearing, sex separation, sterilization (by irradiation or *Wolbachia*), confirmation of mating competitiveness, packaging and transportation, releases and field monitoring [11, 12].

One of the most important steps is the sex separation, ideally at the embryonic stage or at least at the pupal stage, due to the fact that inadvertent female release may affect the success of population suppression by either SIT or IIT programmes and would not be acceptable to the public [8, 13]. For IIT, the inadvertent release of *Wolbachia*-infected females might also lead to population replacement [7, 8, 14, 15]. Since there is no perfect sex separation system or genetic sexing strain (GSS) available for *Ae*. *albopictus* [13], the combination of SIT with IIT is now considered to be the safest approach to suppressing an *Ae*. *albopictus* population [7, 8]. By combining SIT with IIT, the sterility of released males would be maintained via both irradiation and *Wolbachia* infection, while the irradiation would have also caused complete female sterility [7, 8, 14, 15].

A new triple *Wolbachia*-infected (*w*AlbA, *w*AlbB and *w*Pip) *Ae*. *albopictus* strain (known as HC) [16] has been developed by microinjection and complete cytoplasmic incompatibility (CI) was observed between the transinfected males and the wild type females. This HC strain was considered to be suitable for mass rearing with no significant fitness cost observed to be caused by the new transinfection with *w*Pip [16]. In support of the planned combination of SIT and IIT to prevent inadvertent release of fertile females, the minimal irradiation dose to completely sterilize females by irradiation at the pupal stage was shown to be 28 Gy [17]. Moreover, population replacement experiments carried out in small cages indicated that the *w*Pip *Wolbachia* strain could not be spread into a naturally double *Wolbachia*-infected GUA population by introduction of irradiated HC females [17], supporting the hypothesis that irradiation will protect against accidental population replacement. Based on these results, it seems that irradiation will be an effective way to reduce the risk of population replacement instead of population suppression while applying IIT using *Wolbachia* in the absence of a perfect sex separation system [7, 8, 13, 15, 17].

Another important factor in the success of any large scale suppression programme is the ability of laboratory mass-reared and sterilized males to compete with wild males to mate with wild females [18]. Although no standard protocol has been developed for mosquitoes, unlike in other pest species [19], the robustness and ability of colony reared, sterilized (by *Wolbachia*/irradiation or both), and released males to compete may be estimated in the laboratory by measuring parameters such as longevity, size or sperm production [20–22]. Their actual performance in the field may depend more on factors such as flight ability and participation in compatible mating behaviors [23, 24], which are more difficult to measure in the laboratory. All of these parameters could be affected by every procedure of the production process. Many experiments looking at the effect of radiation dose on male mating competitiveness and the effect of release ratio on induced sterility in *Anopheles* [25–28] and *Aedes* [29–31] species have been performed in laboratory and semi-field conditions, and these studies indicate that a balance must be struck between the level of sterility and male mating competitiveness [15, 26, 30].

In *Ae*. *albopictus* SIT, the application of 35 Gy to produce partially sterile males (approx. 96% sterility) is commonly accepted rather than achieving complete sterility at 40 Gy with the corresponding decreased mating competitiveness index [29–31]. However, by combining SIT and IIT strategies to control *Ae*. *albopictus*, the application of a lower radiation dose (28 Gy) in the release generation would completely sterilize any remaining HC females and prevent population replacement, whilst inducing complete sterility in males through the combined irradiation and *Wolbachia* transinfection [17]. Even though a dose of 28 Gy can completely sterilize females, whether this radiation dose has an influence on the mating competitiveness of HC males is still unknown.

In this study, competitiveness tests were performed to assess the effects of pupal irradiation on the mating competitiveness of HC and GUA males, particularly under semi-field conditions. Firstly, the effects of irradiation on adult emergence rate and longevity of HC and GUA males was assessed in small cages. Second, the sterility induced by HC and irradiated HC males in GUA females was calculated at different release ratio in small cages. Finally, the mating competitiveness of sterile *Ae*. *albopictus* males, non-irradiated HC, irradiated HC (28 Gy) and irradiated GUA(35 Gy) males, were compared at a release ratio of 5:1 sterile males to fertile GUA males in large cages. The present study was performed in support of a feasibility study to assess the potential application of the combination of the SIT and IIT to control *Ae*. *albopictus* populations in Guangzhou, China.

## Materials and Methods

### Ethics statement

Studies on *Ae*. *albopictus* mosquito species do not require a specific permit according to the document 2010/63/EU of the European Parliament and the Council on the protection of animals used for scientific purposes. All mosquito strains used in the present study were maintained in the biosecure insectaries of the Joint FAO/IAEA Insect Pest Control Laboratory (IPCL), Seibersdorf, Austria. All the experiments were performed based on standard operating procedures in the IPCL. People who entered into the large cages to collect the females were volunteers and protected well to prevent female biting. The blood used for routine blood-feeding of mosquitoes was collected in Vienna, Austria during routine slaughtering of pigs or cows in a nationally authorized abattoir (Rupert Seethaler, Himbergbei Wien) at the highest possible standards strictly following EU laws and regulations.

### Mosquito strains, rearing and irradiation procedure

Two strains of *Ae*. *albopictus* were used in this experiment: the F_11_ generation of the triple *Wolbachia*-infected (*w*AlbA, *w*AlbB and *w*Pip) HC strain [16] and the F_12_ generation of the naturally double *Wolbachia*-infected GUA strain (*w*AlbA and *w*AlbB). These two strains have a highly similar genetic background due to six generations of backcrossing (F_1_-F_6_) between HC females and GUA males, which originated from Guangzhou, China [16]. The rearing conditions and methods of both HC and GUA strains were as in our previous studies [16, 17], and the strain remains stable in infection status and performance. Mosquitoes used for small and large cage experiments were of the same generation and reared according to the same standard protocol, though performed at a different time, to allow for comparison of results.

In this experiment, irradiation was performed at the pupal stage using a Rad Source RS 2400 X-ray irradiator (Rad Source Technologies Inc., Suwanee, GA) as previous described [17, 27]. Before irradiation, males and females were sexed under the stereoscope at the pupal stage (24–36 hours old). All HC males were irradiated at 28 Gy (IHC) for both small and large cage experiments while the GUA males were irradiated at 35 Gy (IGUA) in large cage experiment. A dose of 35 Gy is proposed for *Ae*. *albopictus* SIT programmes to induce approximately 96% sterility and maintain high male mating competitiveness [29–31]. In contrast, 28 Gy is the minimum dose required to completely sterilize HC females and prevent spread of *Wolbachia* strain *w*Pip, as proposed for combined SIT-IIT programmes, while IHC males are completely sterile due to the irradiation and transinfected *Wolbachia w*Pip strain [17]. All males used in this experiment were three to four days post-emergence and all the virgin GUA females were two to three days post-emergence.

### Effects of irradiation on male emergence and adult longevity in small cages

To assess the effects of irradiation on male emergence and adult longevity (in the absence of female adults), 50 IHC and 50 IGUA male pupae were placed in each of three small cages (0.3 × 0.3 × 0.3 m, 0.027 m^3^, BugDorm 1, MegaView, Taichung, Taiwan) for emergence. Forty eight hours later, the dead pupae were recorded and removed from the cages. Sugar solution (10%) was constantly provided. Dead adults were recorded and removed every day. Three replicates were performed of this experiment. Non-irradiated HC or GUA males were used as controls.

### Sterility induced by HC and IHC males in small cage

The male mating competitiveness of HC and IHC males were examined by testing three experimental ratios (1:1, 5:1, and 10:1) of sterile to fertile males in the small cages. Fifty GUA males (fertile) and 50 virgin GUA females were used in all treatment cages. Control cages with virgin GUA females paired with either only sterile males (HC or IHC males) or only fertile GUA males were also included.

In the experimental cages, either the HC or IHC males together with the GUA males were firstly added into the small cages to acclimatize for one hour before the virgin GUA females were introduced. Competitive mating was then allowed for two days and a 10% sugar solution was provided for the duration of the experiments. After two days, GUA females were collected by aspirator and transferred to new cages. Blood meals were provided to the GUA females and each gravid female was individually placed in a tube for oviposition. Eggs from individual females were matured in adult rearing room for seven days, then hatched, and fecundity and fertility were assessed individually as previously described [16, 17]. Three replicates were performed each for both HC and IHC treatments.

### Induced sterility and mating competitiveness of HC, IHC and IGUA males in large cages

Semi-field experiments were conducted in large cages (1.75 × 1.75 × 1.75 m, 5.36 m^3^, Live Monarch, Boca Raton, Fl.) in a greenhouse setting. Each large cage contained a tray (40 × 29 × 8 cm) containing one liter deionized water as a resting site and one cup containing 80 ml sugar solution (10%) with a piece of coffee filter paper for feeding during the mating period. Experiments in the large cages were conducted in a climate-controlled greenhouse on site at the IPCL under natural light conditions from April to May 2014. Mean temperature in the greenhouse fluctuated between 23 and 26°C with a relative humidity of approximately 50%.

Mating competitiveness of three groups of sterile males was examined in the large cages: HC, IHC and IGUA males. The control cages were performed as described above, with either 50 fertile GUA males or 50 sterile males (HC, IHC or IGUA strains) and 50 GUA females. All competitiveness tests were carried out at a 5:1 sterile: fertile release ratio. Briefly, 250 sterile and 50 fertile males were mixed in a small cage, then taken to the greenhouse for introduction into the large cages. One hour later, 50 virgin GUA females were also introduced into each cage. After two days’ competitive mating, the GUA females were recollected by mouth aspirator. The recollected females were transferred to a new small cage and then maintained under laboratory conditions, as above. Blood meals were provided and the fecundity and fertility assessed as described above. Since the greenhouse could only hold ten cages, this experiment was conducted on two occasions; each time included one cage for the fertile control (GUA males × GUA females), three cages for the sterile control (HC/IHC/IGUA males × GUA females) and two cages for the competitive mating experiment of each type of sterile male. Consequently, two replicates for control cages (both fertile and sterile) and four replicates for experimental cages were completed in this experiment.

### Data collected and statistical analysis

To assess the effect of sterile male releases on a cage’s fertility, the induced sterility (IS) value was calculated as 100% minus the residual fertility value [27], which was calculated from Ho / Hn, where Ho was the observed egg hatch rate from each experimental cage and Hn was the hatch rate from eggs of GUA females mated with fertile males. Male mating competitiveness index (C) was calculated as: C = [(Hn—Ho) / (Ho—Hs)] * (N / S); where Hs were the hatch rate from eggs of GUA females mated with sterile males. Hn and Ho were as described above, and N and S were the numbers of fertile and sterile males, respectively [32]. In this experiment, the IS value was both calculated in small cages in different release ratios and in large cages at 5:1 release ratio. The C value was calculated in different large sterile treatment cages as sterile males (HC/IHC/IGUA) in comparison with fertile males (GUA).

In this experiment, eggs were considered to be from incompatible crosses (HC/IHC/IGUA males × GUA females) when the egg hatch rate was less than the higher Standard Error (SE) of the mean fertility of the sterile control cage. Compatible crosses were divided into two groups. Those egg batches with hatch rate higher than the lower SE of the mean fertility of fertile control cage were considered to be from fertile crosses (GUA males × GUA females). An intermediate egg hatch rate, i.e. fertility between the higher SE of the sterile control mean and lower SE of the fertile control mean, was considered to be from an indeterminate cross [31]. Based on the observed mean egg hatch rate from fertile and sterile control cages, plus or minus their respective SE, individual egg batches were divided into those resulting from copulations with a fertile or a sterile male.

The statistical analysis was conducted using SPSS 13.0 and GraphPad Prism 6.0 software. Normality of all data was examined by the D’Agostino-Pearson omnibus normality test. In our previous study [16], the triple *Wolbachia*-infected HC strain showed no difference in adult emergence or longevity to the double *Wolbachia*-infected GUA strain, so in this experiment when we compared the effects of irradiation on male adult emergence and longevity between HC, IHC, GUA and IGUA strains, we made an assumption that HC and GUA were comparable and only observed the effects of irradiation. The male adult emergence rate was arcsin transformed and comparison between HC, IHC, GUA and IGUA groups was conducted by One-way analysis of variance (ANOVA) and the Tukey hoc post-test. Kaplan-Meier survival analyses (Log rank statistics by Pairwise over strata) were conducted to determine the impact of irradiation on male longevity of HC and GUA strains. For all the experimental and control cages, under laboratory and semi-field conditions, the mean egg hatch rate and IS value of each replicate was arcsin transformed. ANOVA analysis and Tukey hoc post-test were used to compare the differences in mean egg hatch rate, IS value, and C value. In this experiment, females which laid less than 10 eggs were excluded from data analysis.

## Results

### Effects of irradiation on male emergence rate and adult longevity in small cages

No significant difference was observed in the adult emergence rate (Mean ± SD) between HC, IHC, GUA or IGUA males (F = 0.81, df = 3, P>0.05): 98.0 ± 1.2%, 99.3 ± 0.7%, 98.7 ± 0.7% and 98.0 ± 0.1%, respectively.

**Fig 1:**
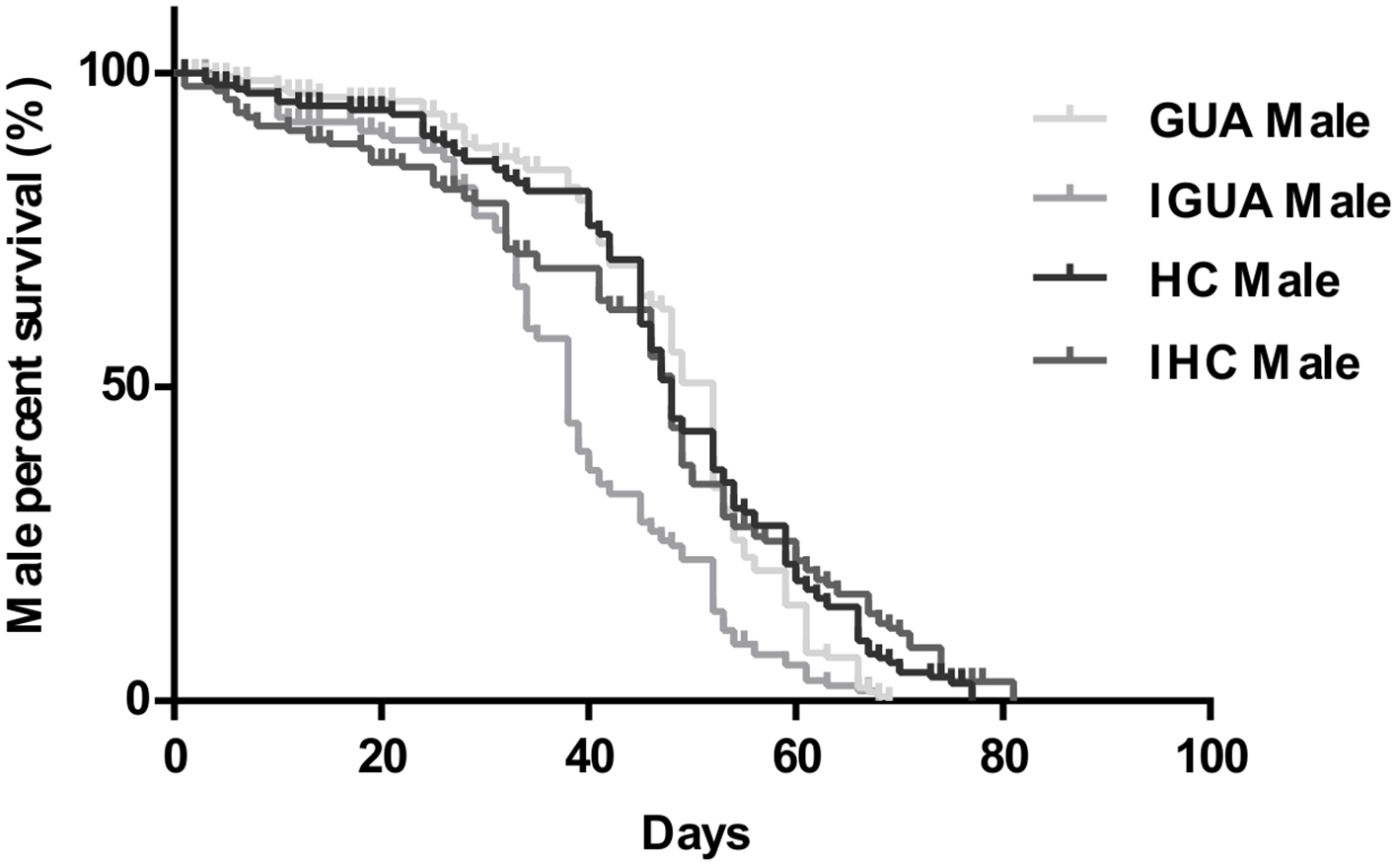
Adult survival curves for the *Aedes albopictus* HC/IHC, and GUA/IGUA males. Day number indicates time post-emergence. Kaplan-Meier curves were used to estimate male adult survival.

No significant difference was observed in survival in the first 7 and 30 days post-emergence between HC, IHC, GUA and IGUA males (data not shown), however, there was a significant difference in average longevity of male adults (χ² = 47.41, df = 3, P<0.05) (Fig 1). In detail, no significant difference was observed in mean longevity between HC, IHC and GUA males (Log rank statistics, P>0.05), however, IGUA males had a significantly shorter lifespan than HC, IHC or GUA males (Log rank statistics, P<0.05). The mean longevity of males was 47.48 ± 1.31d for HC, 44.84 ± 1.70d for IHC, 47.31 ± 1.08d for GUA and 38.10 ± 1.18d for IGUA.

### Sterility induced by HC and IHC males in small cage populations

The fertile control cages (0:1) of both (HC: GUA) and (IHC: GUA) had the highest mean egg hatch rate, and mean fertility significantly decreased as the release ratio of sterile to fertile males increased ((HC: GUA): F = 306.28, df = 4, P<0.05; (IHC: GUA): F = 253.88, df = 4, P<0.05) (Table 1). The egg hatch rate of the sterile control cages (1:0) was (0.5 ± 0.2%, 95% Confidence interval, CI) for the (HC: GUA) cage and (0.2 ± 0.2%, 95% CI) for the (IHC: GUA) cage, which indicated that HC males exhibited a strong CI when mating with the GUA females, whether or not they were also irradiated with 28 Gy (Table 1). In the (HC: GUA) cage, the mean fertility with a release ratio at 1:1 cage was significant higher than at either 5:1 or 10:1 ratios (Tukey hoc post-test, P>0.05), however, no significant difference was found in mean fertility between 5:1 and 10:1 ratio cages (Tukey hoc post-test, P>0.05). However, in the (IHC: GUA) cages, at these three release ratios each cage with a lower release ratio had a higher mean fertility (Table 1).

**Table 1:** Residual fertility and induced sterility of HC and IHC *Aedes albopictus* males at different release ratios in the small cages.

Male: Male	Ratio (Sterile: Fertile)	Fertility (%) (Mean ± 95% CI) (Number of eggs estimated)	IS (%) (Mean ± SD)
**HC: GUA**	0:1	88.1 ± 6.0 (3810) a	-
	1:1	52.9 ± 11.4 (4019) b	39.8 ± 6.1 a
	5:1	16.2 ± 3.7 (3793) c	81.6 ± 1.9 b
	10:1	10.7 ± 8.4 (4215) cd	87.8 ± 4.5 b
	1:0	0.5 ± 0.2 (4451) d	-
**IHC: GUA**	0:1	90.3 ± 4.3 (2887) A	-
	1:1	62.0 ± 13.6 (3397) B	31.3 ± 6.9 A
	5:1	26.7 ± 11.8 (3413) C	70.5 ± 6.0 B
	10:1	9.6 ± 7.3 (3442) D	89.3 ± 3.8 C
	1:0	0.2 ± 0.2 (2768) D	-

CI: Confidence interval.

Within column, values followed by different lowercase letters or capital letters were statistically different in the same treatment cage using ANOVA analysis and Tukey hoc post-test (P<0.05). Three replicates were performed of all treatments.

IS: Induced sterility, calculated as 100% minus the residual fertility value, which was calculated from Ho / Hn, where Ho was the observed egg hatch rate from each experimental cage and Hn was the hatch rate from eggs of GUA females mated with fertile males.

The mean induced sterility (IS) of (HC: GUA) and (IHC: GUA) cages at different release ratios is shown in Table 1. The IS in the (HC: GUA) cage with a 1:1 release ratio was significantly lower than either 5:1 or 10:1 ratio cages (F = 82.6, df = 2, P<0.05), but the IS was not significantly different between the latter two ratios (Tukey hoc post-test, P>0.05) (Table 1). In (IHC: GUA) cages, a significant difference was observed on the IS between each release ratio (F = 73.0, df = 2, P<0.05) (Table 1).

### Induced sterility and mating competitiveness of HC, IHC and IGUA males in large cages

More than 80% (>40) of GUA females could be re-collected by aspirator from each large cage in this experiment.

The egg hatch rate of fertile control cages (GUA males × GUA females) and the three sterile control cages (HC/IHC/IGUA males × GUA females) is shown in Table 2. As in the small cage experiment, almost complete sterility was induced when the HC and IHC males mated with the GUA females, while partial residual fertility (approx. 3.4%) was observed in IGUA males mated with the GUA females (Table 2). This level of residual fertility is consistent with previous studies [29–31]. For the experimental cages at a 5:1 ratio, significant difference was observed in the mean fertility (Ho) and IS depending on the type of male released (Fertility: F = 7.21, df = 2, P<0.05; IS: F = 6.42, df = 2, P<0.05) (Table 2). Specifically, the (IHC: GUA) cage showed a significant lower fertility and higher IS than (IGUA: GUA) cage, while the (HC: GUA) cage exhibited no difference to either (IHC: GUA) or (IGUA: GUA) cages due to the high variability of the observed fertility and IS in the tested four replicates (Table 2). The C values of these three tested groups of males (HC, IHC and IGUA) were significantly different (F = 4.73, df = 2, P<0.05) with the IHC males (0.94) being more than twice as competitive as IGUA males (0.41) (Table 2). The mean C value of HC strain males (0.67) was not significantly different to that of either IHC or IGUA males due to the high variability of the observed C value in the tested four replicates (Tukey hoc post-test, P>0.05) (Table 2).

**Table 2:** Induced sterility and male mating competitiveness index of HC, IHC and IGUA *Aedes albopictus* males in the large cages at a 5:1 (sterile: fertile) ratio.

Male: Male	Fertility* (%) (Mean/Mean ± 95% CI) (Number of eggs estimated)	IS (Mean ± SD)	C (Mean ± SD)
**Fertile control**	86.0 (85.4–86.5) (2478) (Hn)		
**Sterile Control HC**	0.1 (0–0.2) (2278) (Hs)		
**HC: GUA**	**20.0 ± 13.7 ab (4278) (Ho)**	**76.7 ± 8.7 ab**	**0.67 ± 0.34 ab**
**Sterile Control IHC**	0.1 (0–0.2) (1765) (Hs)		
**IHC: GUA**	**15.7 ± 3.7 a (4836) (Ho)**	**81.7 ± 2.3 a**	**0.94 ± 0.17 a**
**Sterile Control IGUA**	3.4 (2.9–3.9) (2124) (Hs)		
**IGUA: GUA**	**31.7 ± 13.6 b (4325) (Ho)**	**63.1 ± 8.6 b**	**0.41 ± 0.19 b**

*:Two replicates each of the fertile and sterile control cages and four replicates of the experimental cages were performed.

CI: Confidence interval.

Hn: Mean egg hatch rate of fertile control cages; Hs: Mean egg hatch rate of sterile control cages; Ho: Mean egg hatch rate of treatment cages.

IS: Induce sterility, calculated as 100% minus the residual fertility values, which was calculated from Ho / Hn.

C: Male mating competitiveness index, calculated as: C = [(Hn—Ho) / (Ho—Hs)] * (N / S), where N and S were the numbers of fertile and sterile males.

Within column, values followed by different lowercase letters were statistically different using ANOVA analysis and Tukey hoc post-test (P<0.05).

When comparing the induced sterility and male mating competitiveness measured in small and large cages at a 5:1 release ratio (Tables 1 and 2), no significant difference was observed in the mean IS value of (HC: GUA) cages and the mean C value of HC males in these two different cage sizes (IS: F = 0.63, df = 1, P>0.05; C: F = 1.54, df = 1, P>0.05). On the contrary, from Tables 1 and 2 we see that the mean IS value of (IHC: GUA) large cages and the mean C value of IHC males in the large cages showed IHC males to be less fertile and more competitive than when tested in the small cages (IS: F = 12.80, df = 1, P<0.05; C: F = 11.39, df = 1, P<0.05).

**Fig 2:**
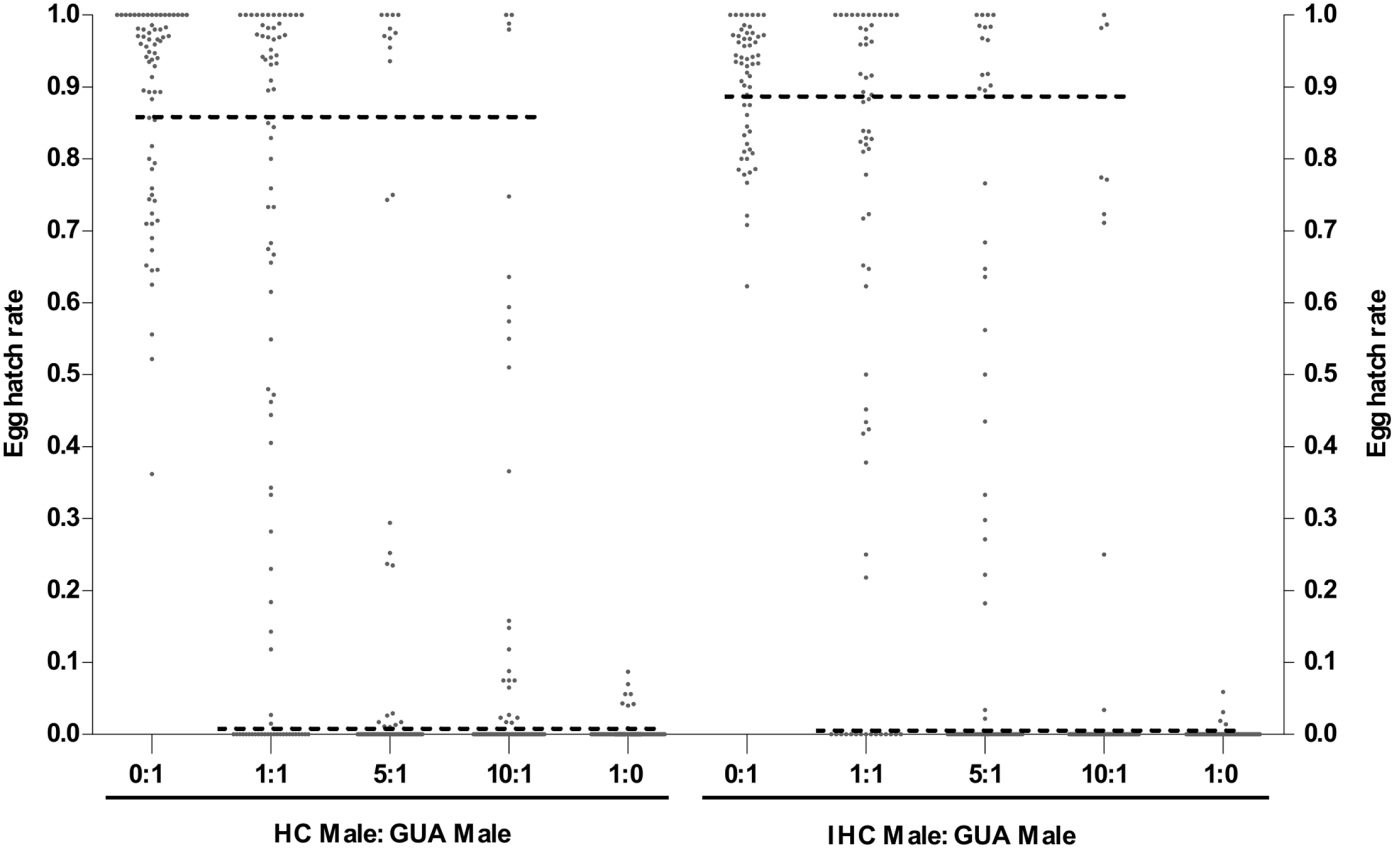
Distribution of egg hatch rates for individual GUA females recollected from experimental cages containing a 1:1, 5:1 or 10:1 ratio of sterile (HC or IHC males) to fertile GUA males, or from control cages containing only sterile or only fertile males in the small cages. The black solid line represents the lower SE of the mean egg hatch rate of either the fertile cages or higher SE of the mean in sterile cages. These limits were used to assign egg batches from competitiveness treatments to either sterile or fertile mating events. Egg batches of an individual female with a hatch rate between the lower SE of the fertile cages and the higher SE of the sterile cages were defined as being intermediate—from an indeterminate mating event.

**Fig 3:**
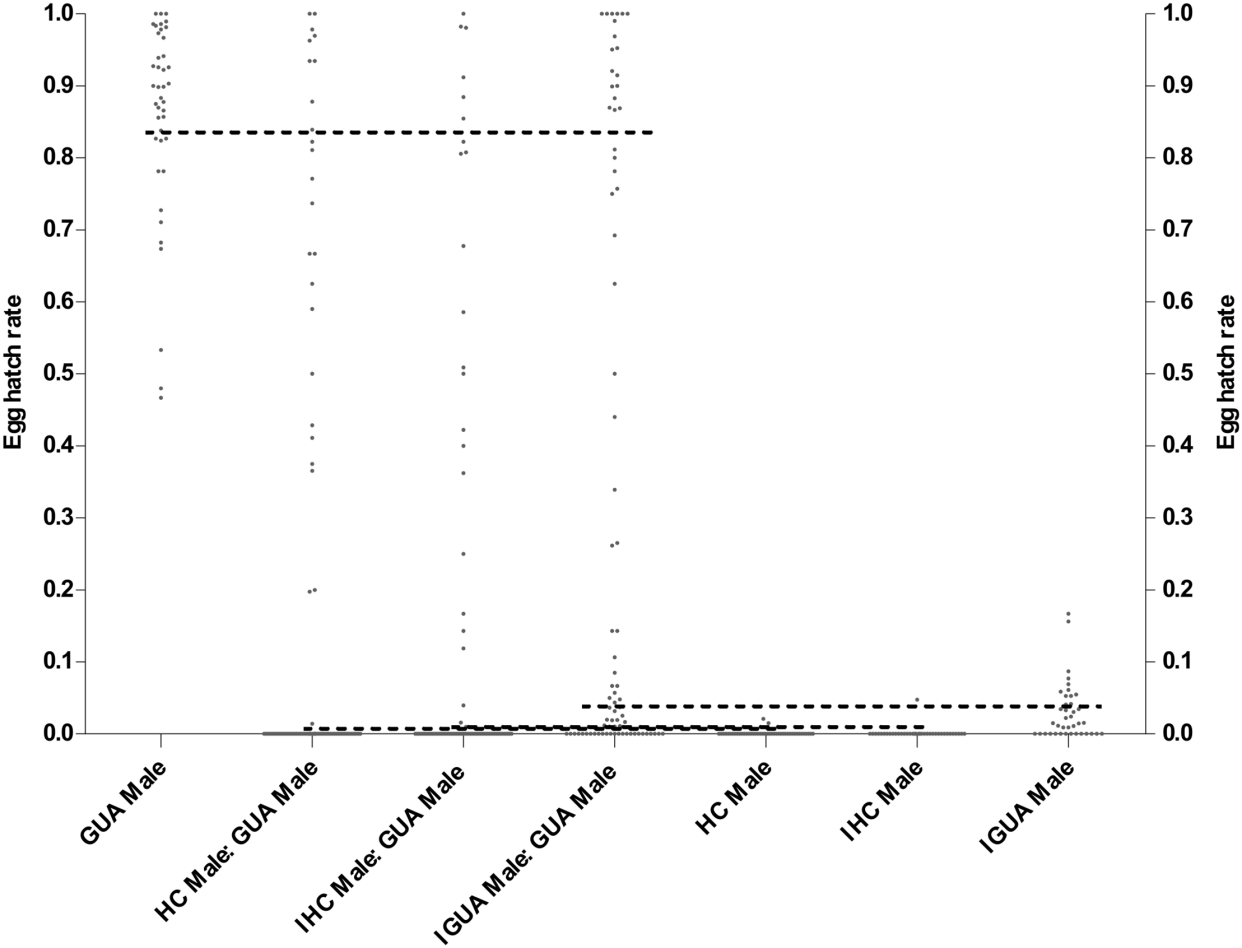
Distribution of egg hatch rates for individual GUA females recollected from experimental cages containing a 5:1 ratio of sterile (HC, IHC and IGUA males) to fertile GUA males or from control cages containing only sterile or fertile males in the large cages. The black solid line represents the lower SE of the mean egg hatch rate of either the fertile cages or higher SE of the mean in sterile cages. These limits were used to assign egg batches from competitiveness treatments to either sterile or fertile mating events. Egg batches of an individual female with a hatch rate between the lower SE of the fertile cages and the higher SE of the sterile cages were defined as being intermediate—from an indeterminate mating event.

Egg hatch rates above the lower SE of the fertile control mean (Small cages: (HC: GUA) = 86.3%, (IHC: GUA) = 89.1%; Large cages: (GUA: GUA) = 83.8%) were defined as being from a fertile copulation; hatch rates below the upper SE of the sterile control mean (Small cages: (HC: GUA) = 0.7%, (IHC: GUA) = 0.3%; Large cages: (HC: GUA) = 0.2%, (IHC: GUA) = 0.2%, (IGUA: GUA) = 4.1%) were defined as being from a sterile copulation (Figs 2 and 3). In the small cages, an indeterminate mating event was inferred from an egg hatch rate of an individual female of between (0.7–86.3%) for (HC: GUA) cages and (0.3–89.1%) for (IHC: GUA) cages (Fig 2). And in the large cages, this event was defined between (0.2–83.8%) for (HC: GUA), (0.2–83.8%) for (IHC: GUA), and (4.1–83.8%) for (IGUA: GUA) cages, respectively (Fig 3). The rate of indeterminate mating events was highest at a small release ratio (1:1) in both (HC: GUA) and (IHC: GUA) small cages ((HC: GUA): 1:1 = 31.0% (26 / 84), 5:1 = 17.1% (13 / 76), 10:1 = 25.0% (20 / 80); (IHC: GUA): 1:1 = 34.9% (22 / 63), 5:1 = 22.5% (16 / 71), 10:1 = 9.1% (6 / 66)). At a 5:1 release ratio in the large cages, the rates of indeterminate mating events were 17.3% (14 / 81) for (HC: GUA), 19.5% (15 / 77) for (IHC: GUA), and 28.9% (22/ 76) for (IGUA: GUA) cages, respectively.

## Discussion

It has been proposed that *Ae*. *albopictus* releases for an IIT programme using the triple *Wolbachia*–infected HC males be irradiated to sterilize any females that could not been removed, to prevent population replacement occurring whilst minimizing any additional impact on male performance. Our previous study confirmed that *Ae*. *albopictus* HC females could be completely sterilized with exposure at the pupal stage to at least 28 Gy [17]. The present study investigated the impact of this irradiation dose on emergence rate, adult longevity and mating competitiveness of HC males under laboratory and semi-field conditions. No negative impacts of 28 Gy of radiation on the adult emergence rate or longevity of HC males were observed under laboratory conditions. By releasing different ratios of HC or IHC males to GUA (sterile: fertile) males in the presence of GUA females, we found that the induced sterility increased with the release ratio in the small cages. Moreover, both HC and IHC males were competitive with the wild type GUA males for mating GUA females in the large cages, which indicated that irradiation of 28 Gy dose had minimal impacts on the male mating competitiveness of the HC strain. Our results indicate that the combination of SIT with IIT strategies might be not only a safe (avoiding the risk of population replacement by accidental releases of transinfected *Wolbachia* females) but also an effective (high male mating competitiveness) approach to control *Ae*. *albopictus* populations.

Adult emergence rate was considered to be an important parameter to measure after pupal irradiation, which is preferable to adult irradiation due to ease of handling and greater robustness [25]. In this study, irradiation had no observable negative impacts on male emergence rate in HC or GUA strains, consistent with our previous finding of high *Ae*. *albopictus* emergence rate (>98%) in irradiated and non-irradiated females even up to a dose of 40 Gy [17]. No difference was observed either in male adult emergence rate (HC *vs* GUA strain) with *Wolbachia* infection status, as in our previous study [16].

Past studies have observed a decrease in male adult longevity after pupal irradiation of *Ae*. *albopictus* at a dose of 40 Gy or higher [27, 33] and in *Anopheles pharoensis* and *An*. *stephensi* irradiated at more than 100 Gy [34, 35]. In this experiment, HC and GUA males were irradiated with 28 and 35 Gy, respectively, at the pupal stage; IGUA males exhibited a reduced longevity compared to HC, IHC and GUA males under the laboratory conditions (Fig 1). In fact, this reduced longevity was only observed later in the lifespan (after 30 days) of IGUA males. This might due to the effects of physical damage caused by irradiation often becoming apparent during the last days of the life of the males [27]. However, it was also interesting that this effect was not seen in older IHC males, on which irradiation seemed to have no impact (Fig 1). Two explanations present themselves for this difference. Firstly, the IHC males were exposed to a lower irradiation dose than IGUA males (28 *vs* 35 Gy). Studies on wild type *Ae*. *albopictus* have found that a higher irradiation dose (>30 Gy) applied to pupae usually leads to a decreased male longevity [27, 33] while a slightly increased longevity after lower-level radiation exposure (20 Gy) was observed by Balestrino et al. (2010) [33]. Secondly, the newly transinfected *w*Pip *Wolbachia* in the HC strain might improve its host’s immunity and reduce damage caused when they were exposed to radiation; however, this requires further investigation. The lack of significant difference in male adult longevity between HC and GUA strains under the laboratory conditions was consistent with our previous study [16].

HC males were found to be competitive with wild type GUA males for mating with GUA females in both small and large cages (Tables 1 and 2). This was consistent with previous studies in which artificially *Wolbachia*-infected mosquitoes were almost equally competitive as non-infected counterparts, due to the only minor fitness cost of *Wolbachia* on their male hosts [36–42]. However, a reduction in male mating performance was observed on the single *w*Ri-infected *Ae*. *albopictus* [43]. The decreased male mating competitiveness (comparison of the observed egg hatch rate with theoretical expectations) might be due to the high fitness cost by *w*Ri strain [43]. For the HC strain used in this experiment, we have already shown that *w*Pip transinfection did not impose any observable reduction in robustness [16].

The performance measured in irradiated males may be affected by the stringency of the test used to compare strains; in larger, more complex or stressful environments weaknesses may be seen which are not apparent in small laboratory cages. Helinski et al (2008) noticed that the mating competitiveness of *An*. *arabiensis* males irradiated as pupae with a dose of 75 Gy was significant lower in when competition took place in large cages than when in similar experiments in laboratory cages [26]. This might have been due to more subtle effects of irradiation which were not apparent in small cage experiments, such as delayed swarm participation or failure to locate mates and achieve insemination under semi-field conditions [27, 28, 44]. Other studies have looked for but failed to observe any difference in male performance based on cage size [30–31]. Surprisingly, our study found that IHC males exhibited a better mating performance under semi-field conditions than in laboratory conditions when released at a 5:1 release ratio, when the same generation was tested after rearing in standard conditions, albeit at a different time (Tables 1 and 2), which requires further investigation.

In both laboratory and large cage experiments some individual female egg batches could not be defined as either sterile or fertile using the criteria chosen, having intermediate hatch rates (Figs 2 and 3). One explanation for this is that these females had mated both a fertile and a sterile male, and laid a mixed egg batch. It used to be thought that wild *Ae*. *aegypti* females only receive one insemination in their lifetime [45, 46], however, recent studies have found that *Aedes* females can receive a second mating both under laboratory [31, 36, 38, 47, 48] and field conditions [49]. A study into multiple inseminations in a single infected (*w*Pip) *Ae*. *albopictus* [38] manipulating the mating sequence (fertile male first and incompatible male after or vice versa) indicated that incompatible males could decrease the fertility of eggs produced by females even if she had first been inseminated by a fertile male. Similarly, when fertile males mated with females after an incompatible male, egg hatch rate could be partially recovered (from 0 to 10%). This may be the explanation for the intermediate hatch rates of some females, beyond the natural range of females mated to fertile males, though further investigation would be required to confirm this. Our study found that the rate of indeterminate crosses *Ae*. *albopictus* in all the large cage treatments was between 17.3 and 28.9%, which was similar to a previous study on *Ae*. *aegypti* which showed that 14% of females could receive a second insemination in tests performed in large enclosures [48].

In this study, both the HC and IHC males were highly competitive with the GUA males under greenhouse conditions, however, a lower male mating competitiveness was observed in the IGUA strain (Table 2), which was contrary to the prior study by Madakacherry et al. (2014) who observed that *Ae*. *albopictus* irradiated as pupae with 35 Gy were equally competitive as non-irradiated counterparts in the same small and large cages [31]. The differences between these two studies might be because the age at which pupae were irradiated was different (24–36 hours old in our study *vs* 40–52 hours old in Madakacherry et al. (2014)). The older pupae when they are irradiated, the better their mating performance is observed [26].

Although it is certainly true that contained experiments cannot truly predict the performance of males released into the wild, the high level of male mating competitiveness when tested in large cages and in fairly natural conditions is encouraging for use of the HC strain, and forms a sound basis for planning an operational suppression programme to test the strain in open release. A competitiveness which is lower than the wild males can be compensated for to an extent by increasing the release ratio, within limits of practical feasibility. For example, in Guatemala, releases of 100:1 sterile: wild male Mediterranean fruit flies (*Ceratitis capitata*) resulted in a significant decline in fertility of a wild population [50]. A release ratio of between 7:1 and 10:1 of sterile to wild male tsetse fly (*Glossina palpalis gambiensis*) was indicated to be competitive to wild type males for mating with target females in Burkina Faso [51]. The current results indicate that *Ae*. *albopictus* HC males, with or without irradiation, is competitive with GUA males in large cages, an important assessment to do prior to initiating any operational field application programme. In addition, no negative impact on male emergence rate or male longevity of the HC strain caused by pupal irradiation was observed in the small cages. Further studies, however, should focus on the IHC males’ survival, tested by mark-release-recapture experiments, in the open field environment and the determination of male mating competitiveness between the IHC males and field-collected males in the large cages. Meanwhile, it will also be interesting to learn whether it is possible to eliminate or suppress the cage populations of the wild type GUA strain by releasing IHC males under semi-field conditions. However, our results so far are encouraging, and indicate that the combination of the SIT with IIT is an approach worth considering to control *Ae*. *albopictus* populations.

## Supporting Information

S1 File Raw data supporting male mating competitiveness trials. This file contains all the raw data for this experiment.
(XLSX)
